# Supplementary material for: TLR2 regulates hair follicle cycle and regeneration via BMP signaling
Source: eLife. 2024 Mar 14;12:RP89335. doi: 10.7554/eLife.89335 (PMC10939499; doi:10.7554/eLife.89335)
Supplement: Figure 4—figure supplement 1—source data 1. [file elife-89335-fig4-figsupp1-data1.pptx]

## Slide 1
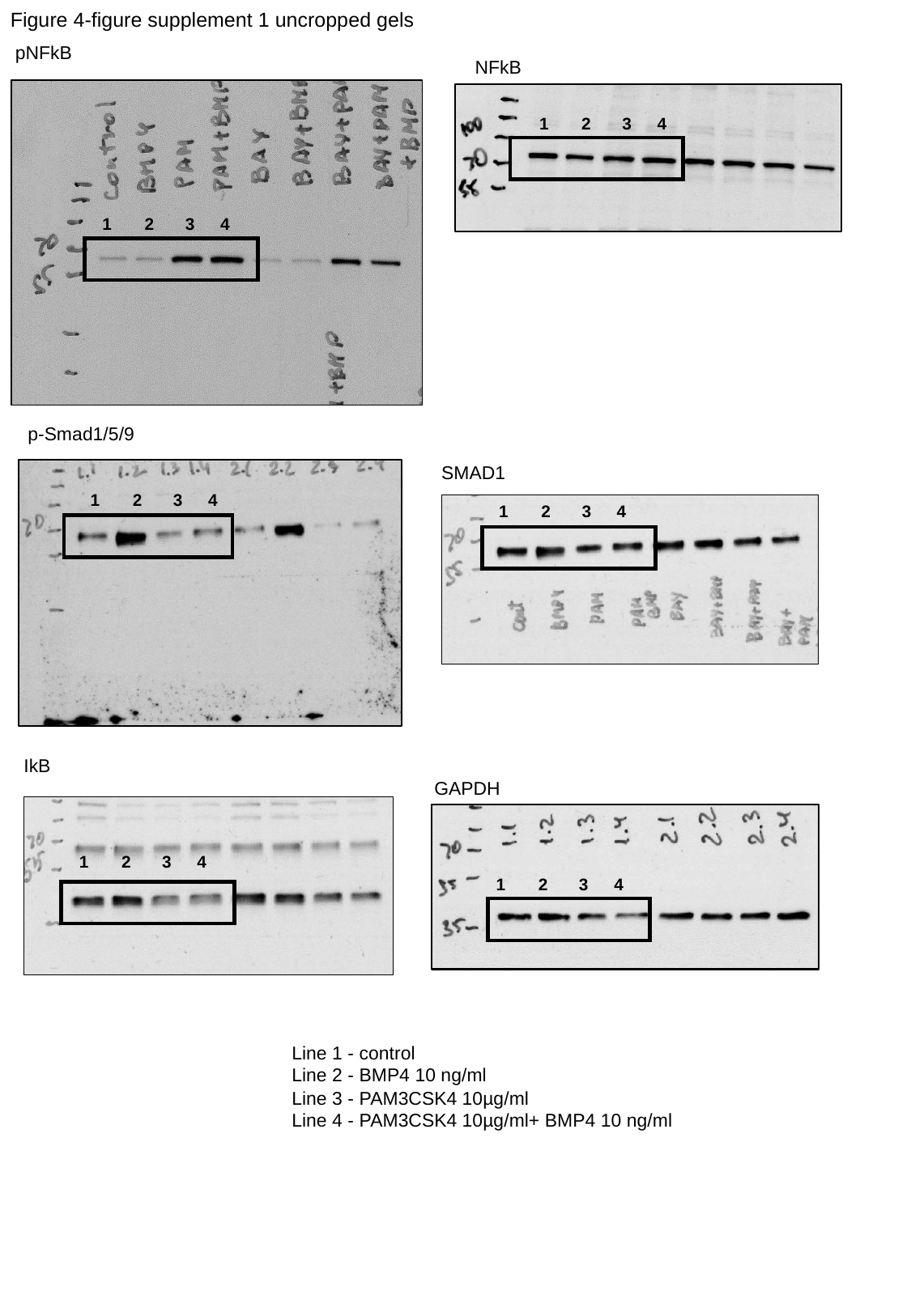

Figure 4-figure supplement 1 uncropped gels
pNFkB
NFkB
1
2
3
4
1
2
3
4
p-Smad1/5/9
1
2
3
4
SMAD1
1
2
3
4
1
2
3
4
GAPDH
1
2
3
4
Line 1 - control
Line 2 - BMP4 10 ng/ml
Line 3 - PAM3CSK4 10µg/ml
Line 4 - PAM3CSK4 10µg/ml+ BMP4 10 ng/ml
