# Supplementary material for: TLR2 regulates hair follicle cycle and regeneration via BMP signaling
Source: eLife. 2024 Mar 14;12:RP89335. doi: 10.7554/eLife.89335 (PMC10939499; doi:10.7554/eLife.89335)
Supplement: Supplementary file 1. [file elife-89335-supp1.docx]

Supplementary File 1

| **TARGET** | **PRIMERS** |
| --- | --- |
| *Tlr2*_F | TCTAAAGTCGATCCGCGACAT |
| *Tlr2*_R | CTACGGGCAGTGGTGAAAACT |
| *Bmp7*_F | ACGGACAGGGCTTCTCCTAC |
| *Bmp7*_R | ATGGTGGTATCGAGGGTGGAA |
| *Bmp2*_F | GGGACCCGCTGTCTTCTAGT |
| *Bmp2*_R | TCAACTCAAATTCGCTGAGGAC |
| *Bmpr1a*_F | AACAGCGATGAATGTCTTCGAG |
| *Bmpr1a* _R | GTCTGGAGGCTGGATTATGGG |
| *Nfkb2*_F | GGCCGGAAGACCTATCCTACT |
| *Nfkb2*_R | CTACAGACACAGCGCACACT |
| *Il1b*_F | GCAACTGTTCCTGAACTCAACT |
| *Il1b* _R | ATCTTTTGGGGTCCGTCAACT |
| *Il6*_F | TAGTCCTTCCTACCCCAATTTCC |
| *Il6*_R | TTGGTCCTTAGCCACTCCTTC |

qPCR primers.
